# Risk Factors for Not Completing a 2-Dose Primary Series of Messenger RNA COVID-19 Vaccination in a Large Health Care System in Southern California: Retrospective Cohort Study

**DOI:** 10.2196/46318

**Published:** 2023-10-04

**Authors:** Stanley Xu, Vennis Hong, Lina S Sy, Katia J Bruxvoort, Bruno Lewin, Bing Han, Kimberly J Holmquist, Lei Qian

**Affiliations:** 1 Department of Research & Evaluation Southern California Permanente Medical Group Pasadena, CA United States; 2 Department of Health Systems Science Kaiser Permanente Bernard J. Tyson School of Medicine Pasadena, CA United States; 3 School of Public Health University of Alabama at Birmingham Birmingham, AL United States

**Keywords:** mRNA COVID-19 vaccines, 2-dose primary series, vaccines, SARS-CoV-2, coronavirus, respiratory, infectious, communicable, immunization, immunize, noncompletion, risk factors, multiple Poisson regression model, COVID-19, vaccination, vaccine, dose, dosing, regression, risk, risks, health outcome, health outcomes, retrospective, cohort, dosage, United States, community, inoculation

## Abstract

**Background:**

COVID-19 vaccination is crucial in combating the COVID-19 pandemic. Messenger RNA COVID-19 vaccines were initially authorized as a 2-dose primary series and have been widely used in the United States; completing the 2-dose primary series offers protection against infection, severe illness, and death. Understanding the risk factors for not completing the 2-dose primary series is critical to evaluate COVID-19 vaccination programs and promote completion of the 2-dose primary series.

**Objective:**

This study examined potential risk factors for not completing a 2-dose primary series of mRNA COVID-19 vaccination.

**Methods:**

We conducted a retrospective cohort study among members aged ≥18 years from a large integrated health care system, Kaiser Permanente Southern California, from December 14, 2020, to June 30, 2022. Noncompletion of the 2-dose primary series was defined as not completing the second dose within 6 months after receipt of the first dose. Crude noncompletion rates were estimated overall and by demographic characteristics, health care use patterns, comorbidity, and community-level socioeconomic factors. A Poisson regression model was fit to examine associations of individual-level and community-level risk factors with noncompletion of the 2-dose primary series.

**Results:**

Among 2.5 million recipients of ≥1 dose of mRNA COVID-19 vaccines, 3.3% (n=81,202) did not complete the second dose within 6 months. Members aged 25-44 years, 65-74 years, and ≥75 years were less likely to not complete the 2-dose primary series than those aged 18-24 years, while members aged 45-64 years were more likely to not complete the 2-dose primary series (adjusted risk ratio [aRR] 1.13, 95% CI 1.10-1.15). Male sex was associated with a higher risk of noncompletion (aRR 1.17, 95% CI 1.15-1.19). Hispanic and non-Hispanic Black race/ethnicity were associated with a lower risk of noncompletion (range aRR 0.78-0.91). Having Medicaid and prior influenza vaccination were associated with a higher risk of noncompletion. Having SARS-CoV-2 infection, experiencing an adverse event, or having an inpatient and emergency department visit during the minimum recommended dose intervals were associated with a higher risk of not completing the 2-dose primary series (aRR 1.98, 95% CI 1.85-2.12; 1.99, 95% CI 1.43-2.76; and 1.85, 95% CI 1.77-1.93, respectively). Those who received the first dose after June 30, 2021, were more likely to not complete the 2-dose primary series within 6 months of receipt of the first dose.

**Conclusions:**

Despite limitations such as being a single-site study and the inability to consider social factors such as employment and vaccine attitudes, our study identified several risk factors for not completing a 2-dose primary series of mRNA vaccination, including being male; having Medicaid coverage; and experiencing SARS-CoV-2 infection, adverse events, or inpatient and emergency department visits during the minimum recommended dose intervals. These findings can inform future efforts in developing effective strategies to enhance vaccination coverage and improve the completion rate of necessary doses.

## Introduction

The COVID-19 pandemic started in December 2019 and has tragically resulted in millions of deaths worldwide. In combating the pandemic, various mitigation measures have been implemented, including social distancing, wearing masks, promoting hygiene practices, testing, and using therapeutic interventions. Among these measures, vaccination has played a crucial role in our collective efforts to combat and control the impact of the COVID-19 pandemic. Among 4 COVID-19 vaccines (BNT162b2, mRNA-1273, Ad26.COV2.S, and NVX-CoV2373) authorized in the United States since December 2020, the 2 (messenger RNA) mRNA COVID-19 vaccines (BNT162b2, manufactured by Pfizer-BioNTech, and mRNA-1273, manufactured by Moderna) have been the most widely used [1]. These COVID-19 vaccines are effective at preventing infection, hospitalization, and death due to SARS-CoV-2 infection [2-5]. Despite these benefits, a major obstacle to COVID-19 vaccination has been vaccine hesitancy. This hesitancy has been linked to concerns regarding vaccine safety, a lack of knowledge about novel COVID-19 vaccines, demographic factors, limited health care access, attitudes toward COVID-19 vaccines, and use and trust in social media [6-11].

Two mRNA vaccines, BNT162b2 and mRNA-1273, were initially authorized as a 2-dose primary series. Optimal protection depends on completing the initial 2-dose primary series of mRNA vaccines according to recommended dose intervals, as well as receiving booster shots to counteract waning immunity and new variants [5,12-19]. As of December 8, 2022, 89.5% (n=231,012,435) of the US population aged 18 years and older received at least 1 COVID-19 vaccine dose, and 76.8% (n=198,386,322) had completed the primary series [1]. Although the vast majority of those who received an mRNA vaccine dose completed the 2-dose primary series, some did not complete the primary series and thus remained underprotected against severe COVID-19 illness and death.

Understanding the risk factors for not completing a 2-dose primary series is critical to evaluate COVID-19 vaccination programs and to promote completion of the 2-dose primary series. Although some studies examined the completion of multidose series of hepatitis A and B vaccines [20,21] and recombinant herpes zoster vaccine [22,23], studies of risk factors associated with not completing a 2-dose primary series of mRNA COVID-19 vaccines are limited. Nguyen et al [24] found associations of sociodemographic characteristics with each of 3 COVID-19 vaccination categories: receipt of at least 1 dose, receipt of the full primary series, and receipt of a booster dose after the primary series. Although sampling methods and data weighting were designed to produce nationally representative results, the sample size was moderate with 74,995 responses from the Household Pulse Survey from December 29, 2021, to January 10, 2022 [25]. Vaccination status for respondents was self-reported and was subject to social desirability bias.

The goal of this study was to examine potential risk factors for not completing a 2-dose primary series of mRNA COVID-19 vaccination in a large health care system in the United States. We considered a range of risk factors including individual-level demographics, clinical comorbidities, prior health care usage, and community-level socioeconomic characteristics, as well as health care usage, adverse events, and SARS-CoV-2 infection during the minimum recommended interval between doses.

## Methods

### Sample and Data

We conducted a retrospective cohort study using electronic health records of members aged ≥18 years from a large integrated health care system, Kaiser Permanente Southern California (KPSC). KPSC serves 4.7 million members of diverse sociodemographic, racial, and ethnic backgrounds at 15 medical centers [26]. To evaluate potential risk factors for not completing a 2-dose primary series of mRNA COVID-19 vaccination, we required members to receive their first dose of either mRNA COVID-19 vaccine from December 14, 2020, to December 31, 2021. We also required members to have continuous membership (allowing up to a 31-day gap) from at least 1 year before to 6 months after the first dose. The study outcome was assessed by June 30, 2022.

### Ethics Approval

Ethics approval for this study was obtained from the KPSC Institutional Review Board On July 5, 2022 (approval 13270). In accordance with 45CFR 46.116, the need for informed consent was waived by the institutional review board because the research activities (secondary analyses of electronic health records data) presented no more than minimal risk to subjects. To protect the privacy and confidentiality of human subjects, all staff working on the research study were trained in procedures to protect the privacy of medical record information. All research data are stored behind a firewall in a password-protected network within the Department of Research & Evaluation at KPSC. Study participants were not compensated given the observational nature of the study.

### Measures of Variables

We assessed the completion rates of 2 mRNA vaccines, BNT162b2 and mRNA-1273. The outcome, noncompletion of the 2-dose primary series, was defined as not completing 2 doses of mRNA vaccination (2 doses of BNT162b2, 2 doses of mRNA-1273 or 1 dose of each) within 6 months after receipt of the first dose. The minimum recommended intervals between dose 1 and dose 2 are 17 days and 24 days (allowing for a 4-day grace period) for BNT162b2 and mRNA-1273, respectively [27,28].

We considered individual-level risk factors including age at receipt of the first dose, sex, race and ethnicity, Medicaid status, health care usage (number of outpatient visits, virtual visits, inpatient visits, and emergency department [ED] visits) within 1 year prior to the first dose date, inpatient or ED visit within 7 days prior to the first dose date (yes or no), Charlson Comorbidity Index within 1 year prior to the first dose date, and receipt of influenza vaccine in the 2 years prior to the first dose date. We also considered community-level risk factors such as neighborhood median household income and neighborhood education level defined as 50% or more than or less than 50% of the neighborhood attaining more than high school education.

Additional individual-level risk factors that were measured during the minimum recommended interval between doses included SARS-CoV-2 infection, encounters with prespecified serious adverse events (AEs), any inpatient or ED visit, and any outpatient visit. SARS-CoV-2 infection was determined by a positive laboratory test for SARS-CoV-2 or a COVID-19 diagnosis. In identifying encounters with serious AEs, 21 types of prespecified AEs were evaluated based on their inclusion as outcomes of interest in COVID-19 vaccine safety studies [29].

In April 2021, COVID-19 vaccinations were expanded from a phased allocation to all eligible adults. To consider the impact of this expansion, we created an indicator for the time of receipt of the first dose of mRNA vaccine before and after June 30, 2021.

### Model and Data Analysis Procedure

Characteristics of completers and noncompleters of the 2-dose primary series were described and compared with chi-square test and 2-tailed *t* test for categorical and continuous risk factors, respectively. To examine the association between potential risk factors and not completing the 2-dose primary series, we fit a multiple Poisson regression model with noncompletion of the 2-dose primary series within 6 months after the first dose as the dependent variable and potential risk factors as the independent variables. The specification of the Poisson regression model is as follows:



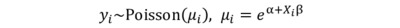



where *µ_i_* is the expected value of *y_i_* given a set of risk factors; *y_i_* is the outcome for the ith subject, *y_i_*=1 for not completing and *y_i_*=0 for completing the 2-dose primary series; α is the intercept; *X_i_* is a row vector of individual- and community-level risk factors, and β is a column vector of corresponding coefficients to be estimated. Because we required members to have continuous membership for at least 6 months after the first dose and the outcome was assessed within 6 months after the first dose, the follow-up period was identical across all individuals; therefore, there was no need to adjust for person time as an offset in the Poisson model. Exponentials of coefficients from the Poisson model can be interpreted as risk ratios.

We reported unadjusted risk ratios (RRs), adjusted risk ratios (aRRs), and 95% CIs. All analyses were conducted using SAS Enterprise Guide (version 8.2; SAS Institute).

## Results

**Figure 1:**
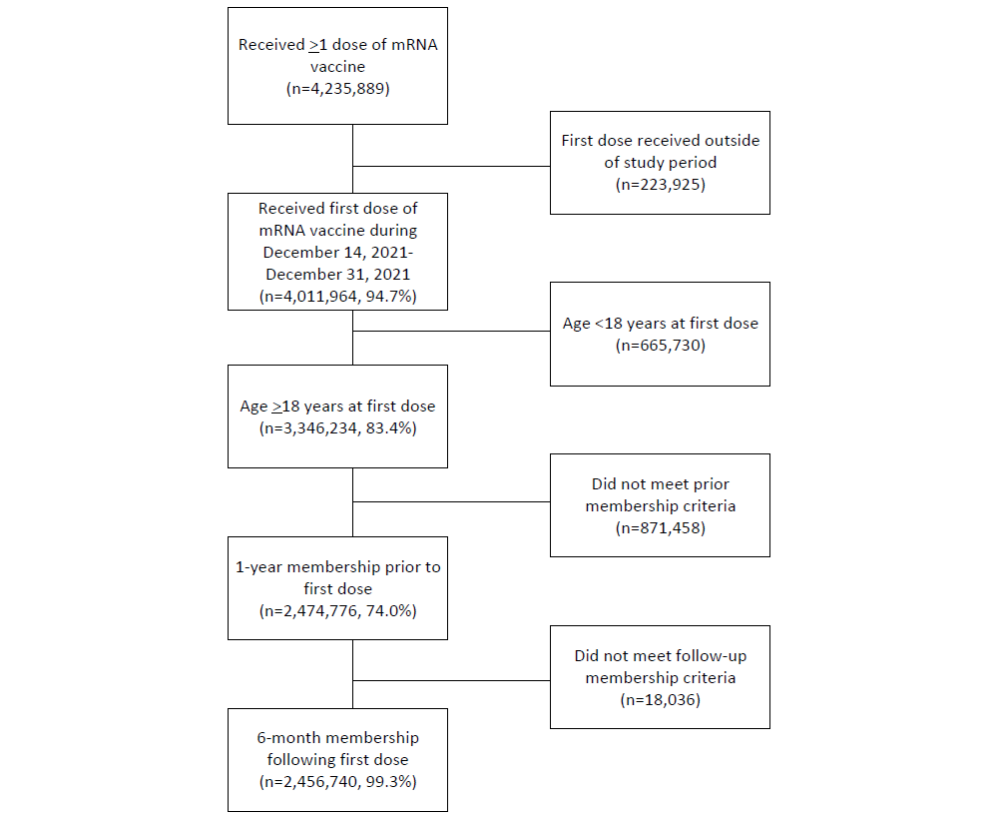
Study population flowchart. mRNA: messenger RNA.

Among more than 4 million members of KPSC who received their first dose of mRNA COVID-19 vaccine during December 14, 2020, to December 31, 2021, 3.3 million (83.4%) were aged ≥18 years (Figure 1). Among members aged ≥18 years who received ≥1 dose of mRNA COVID-19 vaccine, 2.5 million met the membership requirement, and 3.3% (n=81,202) of them did not complete the 2-dose primary series within 6 months after receipt of the first dose (Table 1). Members who were <65 years old, male, or Hispanic were more likely not to complete the primary series. Noncompletion rates were slightly higher among members in communities with a median household income <US $60,000 and lower education level than those in communities with a higher median household income and higher education level. Noncompleters of a 2-dose primary series of mRNA COVID-19 vaccination were less likely to have had influenza vaccine in the 2 years prior to receipt of dose 1 than completers (n=48,886, 60.2% vs n=1,721,833, 72.5%). Noncompleters were more likely to receive the first dose after June 30, 2021, than completers (n=45,216, 55.7% vs n=255,354, 10.8%).

**Table 1:** Characteristics of completers and noncompleters of a 2-dose primary series of messenger RNA vaccination among members of Kaiser Permanente Southern California from December 14, 2020, to December 31, 2021.

Characteristic				Completion of 2-dose primary series within 6 months of first dose					Total (n=2,456,740)			*P* value	
				Yes (n=2,375,538)		No (n=81,202)							
**Age (years), n (%)**													<.001^a^
	18-24		231,728 (9.8)		11,401 (14)		243,129 (10)						
	25-44		748,748 (31.5)		29,126 (35.9)		777,874 (31.7)						
	45-64		818,200 (34.4)		29,658 (36.5)		847,858 (34.5)						
	65-74		350,870 (14.8)		7066 (8.7)		357,936 (14.6)						
	≥75		225,992 (9.5)		3951 (4.9)		229,943 (9.4)						
Age, mean (SD)				49.8 (18.3)		45.4 (17.1)		49.7 (18.2)			<.001^b^		
**Sex, n (%)**													<.001^a^
	Female		1,290,402 (54.3)		39,584 (48.8)		1,329,986 (54.1)						
	Male		1,085,136 (45.7)		41,618 (51.2)		1,126,754 (45.9)						
**Race and ethnicity, n (%)**													<.001^a^
	White		760,981 (32)		24,284 (29.9)		785,265 (32)						
	Hispanic		945,650 (39.8)		33,611 (41.4)		979,261 (40)						
	Asian or Pacific Islander		332,258 (14)		9981 (12.3)		342,239 (13.9)						
	Black		180,116 (7.6)		6321 (7.8)		186,437 (7.6)						
	Other/multiple/unknown		156,533 (6.6)		7005 (8.6)		163,538 (6.7)						
**Number of outpatient visits in 1 year prior to receipt of dose 1, n (%)**													<.001^a^
	0		501,929 (21.1)		20,122 (24.8)		522,051 (21.2)						
	1-4		1044,438 (44)		36,833 (45.4)		1,081,271 (44)						
	5-9		489,910 (20.6)		15,225 (18.8)		505,135 (20.6)						
	≥10		339,261 (14.3)		9022 (11.1)		348,283 (14.2)						
**Number of virtual encounters in 1 year prior to receipt of dose 1, n (%)**													<.001^a^
	0		740,458 (31.2)		32,370 (39.9)		772,828 (31.5)						
	1-4		1,061,283 (44.7)		33,976 (41.8)		1,095,259 (44.6)						
	5-9		350,673 (14.8)		8977 (11.1)		359,650 (14.6)						
	≥10		223,124 (9.4)		5879 (7.2)		229,003 (9.3)						
**Number of inpatient visits in 1 year prior to receipt of dose 1, n (%)**													<.001^a^
	0		2,266,685 (95.4)		77,599 (95.6)		2,344,284 (95.4)						
	1		92,717 (3.9)		2917 (3.6)		95,634 (3.9)						
	≥2		16,136 (0.7)		686 (0.8)		16,822 (0.7)						
**Number of ED^c^ visits in 1 year prior to receipt of dose 1, n (%)**													<.001^a^
	0		2,059,842 (86.7)		69,647 (85.8)		2,129,489 (86.7)						
	1-2		284,371 (12)		10,163 (12.5)		294,534 (12)						
	≥3		31,325 (1.3)		1392 (1.7)		32,717 (1.3)						
**Charlson Comorbidity Index in 1 year prior to receipt of dose 1, n (%)**													<.001^a^
	0		1,669,133 (70.3)		63,373 (78)		1,732,506 (70.5)						
	1		342,218 (14.4)		9757 (12)		351,975 (14.3)						
	2		155,730 (6.6)		3632 (4.5)		159,362 (6.5)						
	≥3		208,457 (8.8)		4440 (5.5)		212,897 (8.7)						
**Had inpatient or ED visit in the 7 days prior to dose 1, n (%)**													<.001^a^
	Yes		9073 (0.4)		487 (0.6)		9560 (0.4)						
	No		2,366,465 (99.6)		80,715 (99.4)		2,447,180 (99.6)						
**Had Medicaid in 1 year prior to receipt of dose 1, n (%)**													<.001^a^
	Yes		165,639 (7)		7453 (9.2)		173,092 (7)						
	No		2,209,899 (93)		73,749 (90.8)		2,283,648 (93)						
**Had influenza vaccine in the 2 years prior to dose 1, n (%)**													<.001^a^
	Yes	1,721,833 (72.5)			48,886 (60.2)		1,770,719 (72.1)						
	No	653,705 (27.5)			32,316 (39.8)		686,021 (27.9)						
**Neighborhood median household income, n (%), US ($)**													<.001^a^
	<$40,000	84,852 (3.6)			3442 (4.2)		88,294 (3.6)						
	$40,000-$59,999	422,975 (17.8)			15,956 (19.7)		438,931 (17.9)						
	$60,000-$79,999	558,709 (23.5)			19,533 (24.1)		578,242 (23.5)						
	$80,000-$99,999	533,012 (22.4)			17,347 (21.4)		550,359 (22.4)						
	≥$100,000	772,181 (32.5)			24,678 (30.4)		796,859 (32.4)						
	Missing	3809 (0.2)			246 (0.3)		4055 (0.2)						
**Neighborhood-level education, n (%)**													<.001^a^
	Less than 50% of the neighborhood attaining greater than a high school education	717,881 (30.2)			26,299 (32.4)		744,180 (30.3)						
	50% or more of the neighborhood attaining greater than a high school education	1,653,747 (69.6)			54,655 (67.3)		1,708,402 (69.5)						
	Missing	3910 (0.2)			248 (0.3)		4158 (0.2)						
**Had SARS-CoV-2 infection during minimum recommended dose interval, n (%)**													<.001^a^
	Yes	5783 (0.2)			858 (1.1)		6641 (0.3)						
	No	2,369,755 (99.8)			80,344 (98.9)		2,450,099 (99.7)						
**Had adverse event during minimum recommended dose interval, n (%)**													<.001^a^
	Yes	410 (0.02)			36 (0.04)		446 (0.02)						
	No	2,375,128 (99.98)			81,166 (99.96)		2,456,294 (99.98)						
**Had inpatient or ED visit during minimum recommended dose interval, n (%)**													<.001^a^
	Yes	35,052 (1.5)			2363 (2.9)		37,415 (1.5)						
	No	2,340,486 (98.5)			78,839 (97.1)		2,419,325 (98.5)						
**Had outpatient visit during minimum recommended dose interval, n (%)**													<.001^a^
	Yes		1,339,444 (56.4)		34,644 (42.7)		1,374,088 (55.9)						
	No		1,036,094 (43.6)		46,558 (57.3)		1,082,652 (44.1)						
**Receipt of first dose after June 30, 2021, n (%)**													<.001^a^
	Yes		255,354 (10.8)		45,216 (55.7)		300,570 (12.2)						
	No		2,120,184 (89.2)		35,986 (44.3)		2,156,170 (87.8)						

^a^Chi-square *P* value.

^b^*t* test *P* value.

^c^ED: emergency department.

Among the 2.5 million members in the study cohort, 6641 (0.3%) members had SARS-CoV-2 infection during the minimum recommended dose interval (Table 1), of whom 858 (12.9%) did not complete the primary series in the 6 months after receipt of the first dose, higher than the overall noncompletion rate of 3.3% (n=81,202). Among 446 (0.02%) members who experienced an AE during the minimum recommended dose interval, 36 (8.1%) did not complete the primary series in the 6 months after receipt of the first dose. Among 37,415 (1.5%) members who had an ED or inpatient visit during the minimum recommended dose interval, 2363 (6.3%) did not complete the primary series in the 6 months after receipt of the first dose.

Unadjusted RRs for noncompletion of the 2-dose primary series are displayed in Figure S1 in Multimedia Appendix 1. The multiple Poisson regression showed that after adjusting for other potential risk factors, members aged 25-44 years, 65-74 years, and ≥75 years were more likely to complete the 2-dose primary series than those aged 18-24 years (aRR for noncompletion 0.94, 95% CI 0.92-0.96; aRR 0.90, 95% CI 0.87-0.93; and 0.92, 95% CI 0.88-0.96, respectively), while members aged 45-64 years were more likely to not complete the 2-dose primary series (aRR 1.13, 95% CI 1.10-1.15) (Figure S2 in Multimedia Appendix 1). Male sex was associated with a higher risk of noncompletion (aRR 1.17, 95% CI 1.15-1.19).

Hispanic and non-Hispanic Black race and ethnicities were associated with lower risk of noncompletion (aRR 0.91, 95% CI 0.90-0.93; aRR 0.78, 95% CI 0.76-0.80, respectively), while Asian and Pacific Islander race was associated with a slightly higher risk of noncompletion (aRR 1.07, 95% CI 1.04-1.09). Having Medicaid in 1 year prior to receipt of dose 1, having influenza vaccine in the 2 years prior to receipt of dose 1, and certain health care use patterns (1-4 and 5-9 outpatient visits, ≥2 inpatient visits, and ≥1 ED visits in the year prior to receipt of dose 1) were associated with a higher risk of noncompletion.

Members with SARS-CoV-2 infection during the minimum recommended dose interval were more likely to be noncompleters (aRR 1.98, 95% CI 1.85-2.12) (Figure S2 in Multimedia Appendix 1). Experiencing an AE during the minimum recommended dose interval was also associated with not completing the primary series (aRR 1.99, 95% CI 1.43-2.76). In addition, members with an inpatient or ED visit during the minimum recommended dose interval were more likely to be noncompleters (aRR 1.85, 95% CI 1.77-1.93), but having an outpatient visit during the minimum recommended dose interval significantly decreased the risk of not completing the primary series (aRR 0.53, 95% CI 0.52-0.53). Receipt of the first dose after June 30, 2021, was associated with a higher risk of not completing the primary series (aRR 9.78, 95% CI 9.63-9.93).

## Discussion

### Principal Findings

Acting in synergy with community pharmacy services and local government mass vaccination efforts, the KPSC health care system was very successful in promoting COVID-19 vaccination and completion of the 2-dose primary series. During the pandemic, KPSC implemented a community-oriented and geographically targeted vaccine strategy aimed at identifying specific zip codes that required additional resources. This proactive approach proved helpful in enhancing COVID-19 vaccination coverage and effectively addressing the disparities in vaccine uptake within underserved communities [30]. Several other aspects should also be considered in designing effective public policy to address future pandemic crises such as Peltzman effects in vaccinations, the emergence of new variants of concern, investment in health care, and the impact of environmental pollution and climate factors [31].

This study showed some impact of sociodemographic characteristics on the completion of a 2-dose primary series of mRNA COVID-19 vaccination in KPSC. For example, after adjusting for other risk factors, the characteristics of being aged ≥65 years, female, and Black or Hispanic, as well as living in a neighborhood with a lower education level, were associated with a lower risk of not completing the 2-dose primary series within 6 months of receipt of the first dose. Members who had ≥2 ED visits in the year prior to receipt of dose 1 were less likely to complete the primary series (aRR for noncompletion 1.23, 95% CI 1.16-1.30). Having SARS-CoV-2 infection, experiencing an AE, or having an inpatient or ED visit during the minimum recommended dose intervals were associated with a higher risk of not completing the 2-dose primary series (aRR 1.98, 95% CI 1.85-2.12; 1.99, 95% CI 1.43-2.76; and 1.85, 95% CI 1.77-1.93, respectively). Those who received the first dose before June 30, 2021, were more likely to complete the 2-dose primary series within 6 months of receipt of the first dose.

In this study, male sex was associated with a higher risk of not completing the 2-dose primary series, while a study by Nguyen et al [24] did not find an association between sex and noncompletion. Although the association of age with noncompletion of the 2-dose primary series in unadjusted analyses (Figure S1 in Multimedia Appendix 1) was largely consistent with those of Nguyen et al [24], the strength of association attenuated after adjusting for other risk factors, from unadjusted RRs of 0.37-0.80 to aRRs of 0.90-1.13. Our finding of lower risk of not completing the 2-dose primary series among Black and Hispanic race and ethnicity was consistent with the study by Nguyen et al [24]; for Asian race and ethnicity, our study found a weak association with not completing the 2-dose primary series (aRR 1.07, 95% CI 1.04-1.09), while Nguyen et al [24] found that Asian race/ethnicity was strongly associated with completion of the 2-dose primary series. Those with lower household income had unadjusted RR ≥1 of not completing the 2-dose primary series (Figure S1 in Multimedia Appendix 1), which is similar to Nguyen et al [24]; however, after adjusting for other risk factors, the association became null. The 2 studies differed in several aspects including the study population (insured vs a general population), study design (retrospective cohort study using electronic health records vs survey study), and risk factors considered. These differences may have contributed to the different findings. In comparison to mRNA COVID-19 vaccination, completion rates for hepatitis A and B vaccinations were observed to be lower, ranging from 40% to 65% across a broad age range [20]. Notably, individuals with Medicaid coverage were found to be associated with a lower completion rate for both the 2-dose primary series of mRNA COVID-19 vaccination in our study and the multidose vaccinations of hepatitis A (2 doses) and B (3 doses) in the study by Nelson et al [20].

With more than 12 months of first-dose data from a large health care organization, this study examined a broad range of potential risk factors including individual- and community-level sociodemographic characteristics, Medicaid status, comorbidities, health care usage, and medical encounters during minimum recommended dose intervals. In a study examining potential factors associated with completing 2 doses of recombinant zoster vaccine among individuals aged ≥50 years, local and systemic reactions after receipt of the first dose were examined in addition to sociodemographic characteristics and health care usage; however, medical encounters with serious AEs were not examined [23]. A unique strength of our study is that we examined the association between medical encounters with serious AEs during minimum recommended dose intervals and noncompletion of the mRNA COVID-19 vaccine 2-dose primary series. Only 87.1% (n=5783) of those who had SARS-CoV-2 infection during the minimum recommended dose intervals completed the primary series, which is significantly lower than the overall completion rate of 96.7% (n=2,375,538). The noncompleters might have felt that it was not necessary to get the second dose because of natural immunity gained from infection. Only 8.1% (n=36) of those who experienced an AE during the minimum recommended dose intervals did not complete the primary series.

There are some limitations in this study. First, the study population is an insured population from a large health care system. The completion rate of the 2-dose primary series among KPSC members aged 18 years and older is 96.7% (n=2,375,538). In contrast, the national completion rate for the primary series, which includes individuals from various health care providers and uninsured individuals, is notably lower at 76.8% (n=198,386,322). The findings in this study may not be generalizable to other health care systems and uninsured populations. Second, data on other risk factors such as members’ employment were not available. Mandatory completion of a 2-dose primary series for employment would decrease the likelihood of noncompletion. Third, we did not consider the requirement of 3 doses for the primary series among immunocompromised individuals [27]. Finally, while we included SARS-CoV-2 infection during the minimum recommended dose intervals as a risk factor for completing the second dose, the impact of SARS-CoV-2 infection prior to receipt of the first dose was not considered.

### Conclusions

To adequately prepare for the pandemic, it is crucial to attain a sufficient level of vaccination coverage, contributing to herd immunity. This will effectively halt the spread of potential virus, thereby protecting people against severe illness and death [32]. Despite limitations such as a study of a single site of an insured population and inability to consider social factors such as employment and attitude to vaccination, our study identified several risk factors for not completing a 2-dose primary series of mRNA vaccination. These factors include being male; having Medicaid coverage; and experiencing SARS-CoV-2 infection, AEs, or inpatient and ED visits during the minimum recommended dose intervals. These findings can inform future efforts in developing effective strategies to enhance vaccination coverage and improve the completion rate of necessary doses.

## Appendix

Multimedia Appendix 1 Risk ratios (95% CI) of not completing the 2-dose primary series of mRNA COVID-19 vaccination within 6 months of the first dose given among members of Kaiser Permanente Southern California during December 14, 2020 to December 31, 2021.

## Abbreviations

AE: adverse event
aRR: adjusted risk ratio
ED: emergency department
KPSC: Kaiser Permanente Southern California
mRNA: messenger RNA
RR: risk ratio

## Data Availability

Individual-level data reported in this study involving human research participants are not publicly shared due to potentially identifying or sensitive patient information. Upon request to the corresponding author, and subject to review and approval of an analysis proposal, KPSC may provide the deidentified aggregate-level data that support the findings of this study within 6 months. Anonymized data (deidentified data including participant data as applicable) that support the findings of this study may be made available from the investigative team in the following conditions: (1) agreement to collaborate with the study team on all publications, (2) provision of external funding for administrative and investigator time necessary for this collaboration, (3) demonstration that the external investigative team is qualified and has documented evidence of training for human subjects protections, and (4) agreement to abide by the terms outlined in data use agreements between institutions.

## References

[1] (2022). COVID-19 vaccinations in the United States. Centers for Disease Control and Prevention.

[2] Polack FP, Thomas SJ, Kitchin N, Absalon J, Gurtman A, Lockhart S, Perez JL, Marc GP, Moreira ED, Zerbini C, Bailey R, Swanson KA, Roychoudhury S, Koury K, Li P, Kalina WV, Cooper D, Frenck RW, Hammitt LL, Türeci Ö, Nell H, Schaefer A, Ünal S, Tresnan DB, Mather S, Dormitzer PR, Şahin U, Jansen KU, Gruber WC, C4591001 Clinical Trial Group (2020). Safety and efficacy of the BNT162b2 mRNA Covid-19 vaccine. N Engl J Med.

[3] Baden LR, El Sahly HM, Essink B, Kotloff K, Frey S, Novak R, Diemert D, Spector SA, Rouphael N, Creech CB, McGettigan J, Khetan S, Segall N, Solis J, Brosz A, Fierro C, Schwartz H, Neuzil K, Corey L, Gilbert P, Janes H, Follmann D, Marovich M, Mascola J, Polakowski L, Ledgerwood J, Graham BS, Bennett H, Pajon R, Knightly C, Leav B, Deng W, Zhou H, Han S, Ivarsson M, Miller J, Zaks T, COVE Study Group (2021). Efficacy and safety of the mRNA-1273 SARS-CoV-2 vaccine. N Engl J Med.

[4] Magazzino C, Mele M, Coccia M (2022). A machine learning algorithm to analyse the effects of vaccination on COVID-19 mortality. Epidemiol Infect.

[5] Rosero-Bixby L (2022). The effectiveness of pfizer-biontech and Oxford-astrazeneca vaccines to prevent severe COVID-19 in Costa Rica: nationwide, ecological study of hospitalization prevalence. JMIR Public Health Surveill.

[6] Griffith J, Marani H, Monkman H (2021). COVID-19 vaccine hesitancy in Canada: content analysis of Tweets using the theoretical domains framework. J Med Internet Res.

[7] Yuan Y, Melde C, Zhang N, Pagidipati P (2023). Race, ethnicity, psychological factors, and COVID-19 vaccine hesitancy during the COVID-19 pandemic. Psychol Health Med.

[8] Matas JL, Landry LG, Lee L, Hansel S, Coudray MS, Mata-McMurry LV, Chalasani N, Xu L, Stair T, Edwards C, Puckrein G, Meyer W, Wiltz G, Sampson M, Gregerson P, Barron C, Marable J, Akinboboye O, Il'yasova D (2023). Demographic determinants and geographical variability of COVID-19 vaccine hesitancy in underserved communities: cross-sectional study. JMIR Public Health Surveill.

[9] Moon I, Han J, Kim K (2023). Determinants of COVID-19 vaccine hesitancy: 2020 California health interview survey. Prev Med Rep.

[10] Jones LF, Bonfield S, Farrell J, Weston D (2023). Understanding the public's attitudes toward COVID-19 vaccines in Nottinghamshire, United Kingdom: qualitative social media analysis. J Med Internet Res.

[11] Carrieri V, Guthmuller S, Wübker A (2023). Trust and COVID-19 vaccine hesitancy. Sci Rep.

[12] Livingston EH (2021). Necessity of 2 doses of the pfizer and moderna COVID-19 vaccines. JAMA.

[13] Martínez-Baz I, Trobajo-Sanmartín C, Miqueleiz A, Guevara M, Fernández-Huerta M, Burgui C, Casado I, Portillo ME, Navascués A, Ezpeleta C, Castilla J, Working Group for the Study of COVID-19 in Navarre, Investigators‚ other members of the Working Group for the Study of COVID-19 in Navarre (2021). Product-specific COVID-19 vaccine effectiveness against secondary infection in close contacts, Navarre, Spain, April to August 2021. Euro Surveill.

[14] Goldberg Y, Mandel M, Bar-On YM, Bodenheimer O, Freedman L, Haas EJ, Milo R, Alroy-Preis S, Ash N, Huppert A (2021). Waning immunity after the BNT162b2 vaccine in Israel. N Engl J Med.

[15] Tartof SY, Slezak JM, Fischer H, Hong V, Ackerson BK, Ranasinghe ON, Frankland TB, Ogun OA, Zamparo JM, Gray S, Valluri SR, Pan K, Angulo FJ, Jodar L, McLaughlin JM (2021). Effectiveness of mRNA BNT162b2 COVID-19 vaccine up to 6 months in a large integrated health system in the USA: a retrospective cohort study. Lancet.

[16] Bruxvoort KJ, Sy LS, Qian L, Ackerson BK, Luo Y, Lee GS, Tian Y, Florea A, Takhar HS, Tubert JE, Talarico CA, Tseng HF (2022). Real-world effectiveness of the mRNA-1273 vaccine against COVID-19: interim results from a prospective observational cohort study. Lancet Reg Health Am.

[17] Tseng HF, Ackerson BK, Luo Y, Sy LS, Talarico CA, Tian Y, Bruxvoort KJ, Tubert JE, Florea A, Ku JH, Lee GS, Choi SK, Takhar HS, Aragones M, Qian L (2022). Effectiveness of mRNA-1273 against SARS-CoV-2 Omicron and Delta variants. Nat Med.

[18] Korves C, Izurieta HS, Smith J, Zwain GM, Powell EI, Balajee A, Ryder KM, Young-Xu Y (2022). Relative effectiveness of booster vs. 2-dose mRNA Covid-19 vaccination in the Veterans Health Administration: self-controlled risk interval analysis. Vaccine.

[19] Rosenblum HG, Wallace M, Godfrey M, Roper LE, Hall E, Fleming-Dutra KE, Link-Gelles R, Pilishvili T, Williams J, Moulia DL, Brooks O, Talbot HK, Lee GM, Bell BP, Daley MF, Meyer S, Oliver SE, Twentyman E (2022). Interim recommendations from the advisory committee on immunization practices for the use of bivalent booster doses of COVID-19 vaccines - United States, october 2022. MMWR Morb Mortal Wkly Rep.

[20] Nelson JC, Bittner RCL, Bounds L, Zhao S, Baggs J, Donahue JG, Hambidge SJ, Jacobsen SJ, Klein NP, Naleway AL, Zangwill KM, Jackson LA (2009). Compliance with multiple-dose vaccine schedules among older children, adolescents, and adults: results from a vaccine safety datalink study. Am J Public Health.

[21] Bruxvoort K, Slezak J, Huang R, Ackerson B, Sy LS, Qian L, Reynolds K, Towner W, Solano Z, Mercado C, Hyer R, Janssen R, Jacobsen SJ (2020). Association of number of doses with hepatitis B vaccine series completion in US adults. JAMA Netw Open.

[22] Patterson BJ, Chen CC, McGuiness CB, Glasser LI, Sun K, Buck PO (2021). Early examination of real-world uptake and second-dose completion of recombinant zoster vaccine in the United States from October 2017 to September 2019. Hum Vaccin Immunother.

[23] Ackerson B, Qian L, Sy LS, Bruxvoort K, Wu J, Luo Y, Diaz-Decaro J, Talarico C, Tseng HF (2021). Completion of the two-dose recombinant zoster vaccine series in adults 50 years and older. Vaccine.

[24] Nguyen KH, Chen Y, Huang J, Allen JD, Beninger P, Corlin L (2022). Who has not been vaccinated, fully vaccinated, or boosted for COVID-19?. Am J Infect Control.

[25] (2022). Source of the Data and Accuracy of the Estimates for the Household Pulse Survey – Phase 3.3. US Census Bureau.

[26] Koebnick C, Langer-Gould AM, Gould MK, Chao CR, Iyer RL, Smith N, Chen W, Jacobsen SJ (2012). Sociodemographic characteristics of members of a large, integrated health care system: comparison with US Census Bureau data. Perm J.

[27] (2021). Use of COVID-19 vaccines in the United States. Centers for Disease Control and Prevention.

[28] (2023). Interim clinical considerations for use of COVID-19 vaccines in the United States. Centers for Disease Control and Prevention.

[29] Xu S, Hong V, Sy LS, Glenn SC, Ryan DS, Morrissette KL, Nelson JC, Hambidge SJ, Crane B, Zerbo O, DeSilva MB, Glanz JM, Donahue JG, Liles E, Duffy J, Qian L (2022). Changes in incidence rates of outcomes of interest in vaccine safety studies during the COVID-19 pandemic. Vaccine.

[30] Swope M, Alem AC, Russo SC, Gin NE, Chevez SG, Haque R (2023). Developing a community-oriented and place-based strategy to improve COVID-19 vaccine accessibility. Perm J.

[31] Coccia M (2022). COVID-19 vaccination is not a sufficient public policy to face crisis management of next pandemic threats. Public Organization Review.

[32] Coccia M (2022). Optimal levels of vaccination to reduce COVID-19 infected individuals and deaths: a global analysis. Environ Res.

