# Supplementary material for: Risk Factors for Not Completing a 2-Dose Primary Series of Messenger RNA COVID-19 Vaccination in a Large Health Care System in Southern California: Retrospective Cohort Study
Source: JMIR Public Health Surveill. 2023 Oct 4;9:e46318. doi: 10.2196/46318 (PMC10563864; doi:10.2196/46318)
Supplement: Multimedia Appendix 1 [file publichealth_v9i1e46318_app1.pdf]

**Figure S1.** Unadjusted risk ratios (95% CI) of not completing the 2-dose primary series of mRNA COVID-19 vaccination within 6 months of the first dose given among members of Kaiser Permanente Southern California during December 14, 2020 to December 31, 2021.

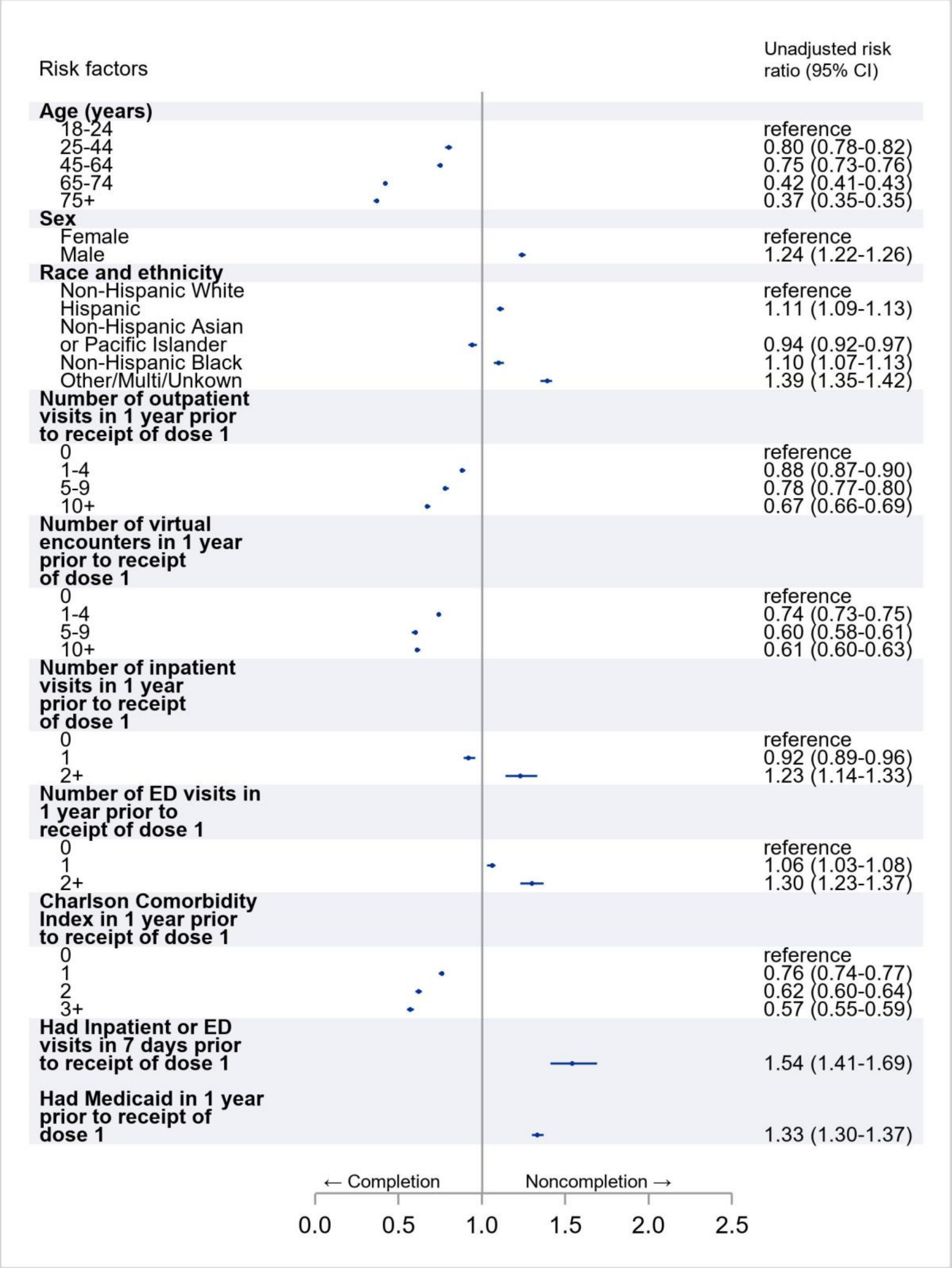

**Figure S1.** (continued)

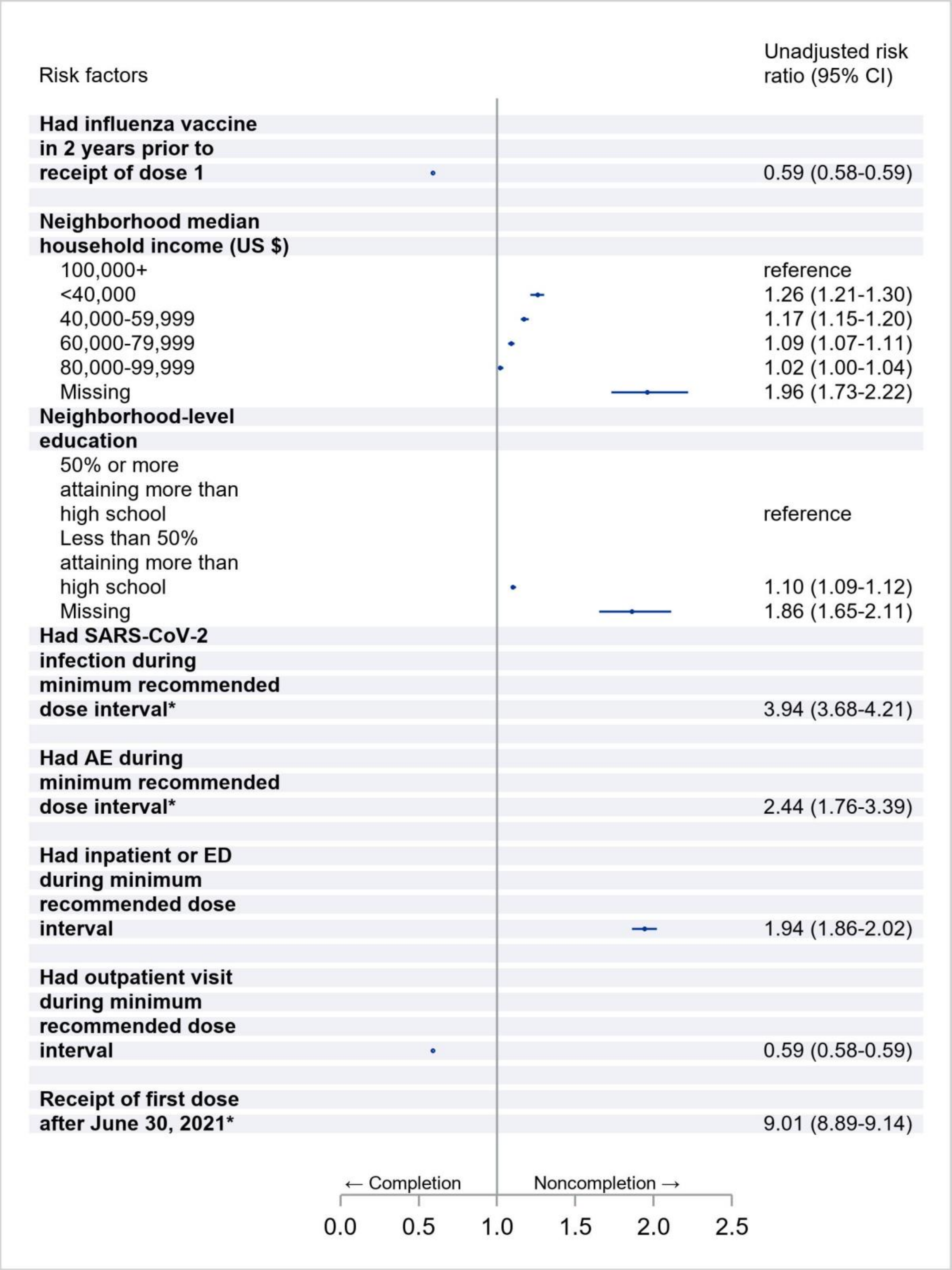


AE, adverse event; ED, emergency department
*Estimates could not be displayed on the plot due to scale limitations

**Figure S2.** Adjusted risk ratios (95% CI) of not completing the 2-dose primary series of mRNA COVID-19 vaccination within 6 months of the first dose given among members of Kaiser Permanente Southern California during December 14, 2020 to December 31, 2021.

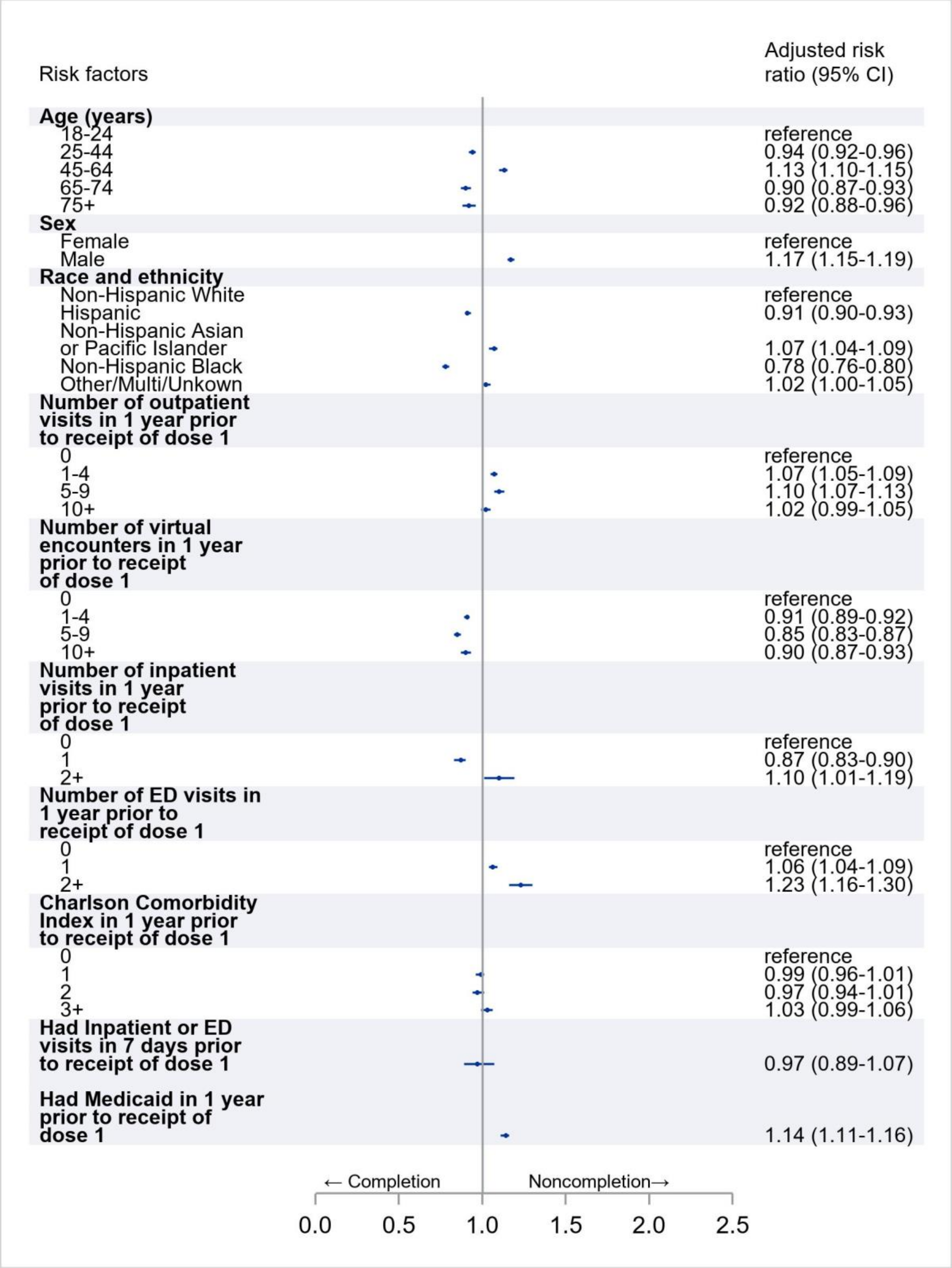

**Figure S2.** (continued)

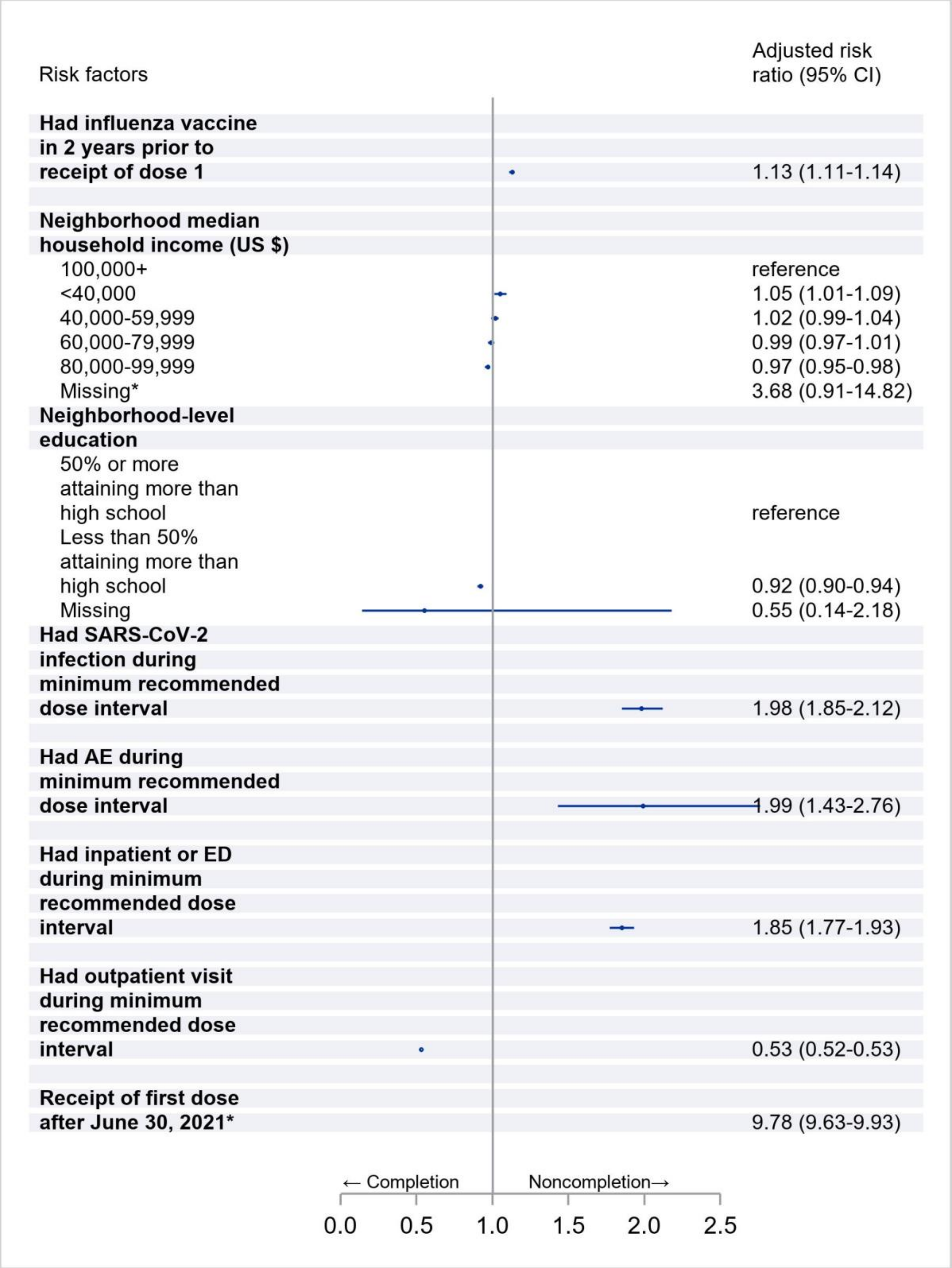


AE, adverse event; ED, emergency department
*Estimates could not be displayed on the plot due to scale limitations
